# Non-Coding RNAs Set a New Phenotypic Frontier in Prostate Cancer Metastasis and Resistance

**DOI:** 10.3390/ijms22042100

**Published:** 2021-02-20

**Authors:** Joshua Altschuler, Jennifer A. Stockert, Natasha Kyprianou

**Affiliations:** 1 Department of Urology, The Tisch Cancer Institute, Icahn School of Medicine at Mount Sinai, New York, NY 10029, USA; joshua.altschuler@mountsinai.org (J.A.); jennifer.stockert@mssm.edu (J.A.S.); 2 Department of Oncological Sciences, Icahn School of Medicine at Mount Sinai, New York, NY 10029, USA

**Keywords:** therapeutic resistance, NEPC, EMT, metastasis, anoikis, ncRNA, miRNA, lncRNA

## Abstract

Prostate cancer (PCa) mortality remains a significant public health problem, as advanced disease has poor survivability due to the development of resistance in response to both standard and novel therapeutic interventions. Therapeutic resistance is a multifaceted problem involving the interplay of a number of biological mechanisms including genetic, signaling, and phenotypic alterations, compounded by the contributions of a tumor microenvironment that supports tumor growth, invasiveness, and metastasis. The androgen receptor (AR) is a primary regulator of prostate cell growth, response and maintenance, and the target of most standard PCa therapies designed to inhibit AR from interacting with androgens, its native ligands. As such, AR remains the main driver of therapeutic response in patients with metastatic castration-resistant prostate cancer (mCRPC). While androgen deprivation therapy (ADT), in combination with microtubule-targeting taxane chemotherapy, offers survival benefits in patients with mCRPC, therapeutic resistance invariably develops, leading to lethal disease. Understanding the mechanisms underlying resistance is critical to improving therapeutic outcomes and also to the development of biomarker signatures of predictive value. The interconversions between epithelial-to-mesenchymal transition (EMT) and mesenchymal-to-epithelial transition (MET) navigate the prostate tumor therapeutic response, and provide a novel targeting platform in overcoming therapeutic resistance. Both microRNA (miRNA)- and long non-coding RNA (lncRNA)-mediated mechanisms have been associated with epigenetic changes in prostate cancer. This review discusses the current evidence-based knowledge of the role of the phenotypic transitions and novel molecular determinants (non-coding RNAs) as contributors to the emergence of therapeutic resistance and metastasis and their integrated predictive value in prostate cancer progression to advanced disease.

## 1. Introduction

### 1.1. Prostate Cancer: The Clinical Challenge

Prostate cancer (PCa) remains among the most significant public health concerns in the United States, predicted to account for approximately 13.09% of all new cancer cases diagnosed and lead to 34,130 deaths in 2021 [1]. It is the third most commonly diagnosed malignancy in the US behind breast and lung cancers [2] and is the most commonly diagnosed cancer in men [3]. Since the advent of prostate specific antigen (PSA) screening, there has been a significant shift in the stage at which patients are diagnosed, as many more men are now discovering and treating their disease while it is still locally confined [4]. While five-year survival rates for patients with localized PCa approach 98–100%, survival rates dramatically decrease once the cancer has metastasized to distant sites [5]. Localized primary PCa is effectively treated with either surgery (radical prostatectomy) or radiation therapy, or a combination of the two [6]. For recurrent or metastatic disease, androgen deprivation therapy (ADT) is the primary standard of care and works by either lowering androgen levels via blocking the production of the hormone centrally, or by inhibiting the binding of androgens to their receptors peripherally [7]. ADT targets the androgen receptor (AR) signaling network that is essential to the proper functioning, growth and maintenance of prostate cells [8]. Androgen deprivation can be achieved surgically (via orchiectomy) or pharmacologically with the chronic administration of luteinizing hormone releasing-hormone (LHRH) agonists [9] or AR blockers such as bicalutamide, flutamide and nilutamide [6]. Although many patients will initially respond to ADT, most patients will eventually develop castration-resistant prostate cancer (CRPC) and ultimately metastatic lethal disease, despite having castrate levels of androgens [10].

### 1.2. Mechanisms of Therapeutic Resistance

The selective pressure that ADT imposes upon treatment-naïve cancer leads to the emergence of the androgen-independent state. Androgen-independent cancer cells rely on a host of alternative, adaptive mechanisms that are designed to compensate for the lack of androgen stimulation to sustain growth [11]. AR-dependent mechanisms span multiple levels of the androgen axis and affect the synthesis, utilization and signaling of androgens. These mechanisms include the altered intratumoral production of androgens, AR amplification and overexpression, AR point mutations, and the expression of constituently-active AR splice-variants [12]. Although the primary effect of ADT is the reduction in androgen levels to castrate levels, it has been shown that patients with CRPC have persistently elevated levels of androgens that may be derived from the production of dihydrotestosterone (DHT) intratumorally [13]. Prostate cancer cells are also able to convert weaker androgens produced by the adrenal glands, such as androstenedione and dehydroepiandrosterone (DHEA), into testosterone and DHT, which have a higher affinity for AR [14]. Importantly, several studies have revealed that even low levels of androgens are enough to significantly amplify AR in vivo [15,16,17] and that amplified AR can, in turn, efficiently utilize and augment this signal [18]. AR sensitivity to low levels of androgens may also be heightened by the presence of coactivators that enhance AR function [19,20]. One study that examined samples of PCa bone metastases from treated patients found that AR was amplified in 53% of samples [21]. In contrast, AR amplification was rarely detected in patients with primary prostate tumors [22]. Furthermore, through promiscuous AR activation, ligands aside from androgens, testosterone and its derivatives can activate AR. This process occurs as a result of mutations in the AR gene which alter the ligand binding domain (LBD) [23]. AR mutations are rare in early disease but are frequently seen in CRPC [24], and these mutations allow for receptor activation by other steroid hormones such as estrogen, progesterone, and glucocorticoids [25,26]. Certain mutations can also provide resistance against the effects of AR inhibitors; for example, the T877A mutation protects AR against a major active metabolite of flutamide, hydroxyflutamide, [11,27] while the W741C/L mutation protects against bicalutamide [11,28]. Coactivator proteins can also increase AR responsiveness to alternative ligands, such as coactivator ARA70 that increases AR sensitivity to estradiol [19,29]. Several growth factors, including insulin-like growth factor 1 (IGF-1) [30], human epidermal growth factor receptor 2 (HER2/neu) [31], keratinocyte growth factor (KGF), and epidermal growth factor (EGF), can also activate AR [19,32]. In addition, interleukins IL-6 and IL-8 are capable of stimulating AR (IL-6) [33,34,35] and promoting AR transcriptional activity (IL-8) [34]. AR splice variants (AR-Vs) represent another major functional adaption of AR and mechanism of resistance to ADT [11,36]. Some of these variants are constitutively active and can transcribe AR target genes in the absence of natural AR ligands [11,19,37].

Evasion of apoptosis control including the activation of PTEN/PI3K/AKT survival signaling is an important contributor and advanced metastatic disease (mCRPC) [11]. PTEN (phosphatase and tensin homolog) is a tumor suppressor that dephosphorylates PIP3 and negatively regulates the phosphoinositide 3-kinase (PI3K) pathway [8] that, when lost, leads to constitutive activation of PI3K, which acts downstream to activate protein kinase B (AKT) and mTOR (mammalian target of rapamycin) signaling [8,38] pathways that are involved in the regulation of cell growth, survival, and proliferation [39] and protein synthesis [38], respectively. Loss of PTEN is a major event in prostate cancer pathogenesis and appears to play a role in the development of CRPC [8,40]. Deletions or inactivating mutations of PTEN have been observed in approximately 20% of primary prostate cancer samples and in over 50% of cases of CRPC [41,42]. While elegant studies by different groups have demonstrated that negative reciprocal feedback between PI3K and AR signaling pathways can act as an AR-independent mechanism to develop CRPC in PTEN-deficient models of PCa [40,43], there is evidence to suggest a functional interplay between the PI3K/AKT pathways and AR, wherein AR and AKT work synergistically to promote PCa initiation and progression [44]. The NF-κB transcription factor signaling pathway also prominently contributes to CRPC, as NF-κB sustains AR activity [11] and significantly increases AR at both mRNA and protein levels [45].

Enzalutamide and abiraterone are the leading second-generation antiandrogens FDA-approved for the treatment of CRPC [46]. Enzalutamide acts by binding to the LBD of AR, and blocks androgen-AR complex nuclear translocation, thereby preventing the binding of this complex to DNA [6]. Abiraterone targets the biosynthesis of testosterone by inhibiting the cytochrome p450 enzyme 17R-hydroxylase-17,20-lyase (CYP17), an enzyme found in the testes and adrenal glands [6]. Several clinical trials (as described in Table 1) have shown the efficacy of these drugs in that enzalutamide improved overall survival (OS) and radiographic progression free survival (PFS) in patients with CRPC who had received chemotherapy [47], as well as in those who were chemotherapy-naïve [48], while abiraterone treatment combined with prednisone improved overall survival in chemotherapy-naïve men with mCRPC [49]. Resistance to both drugs invariably develops, however [50], and is attributed to various mechanisms; thus, a missense mutation in the LBD of AR, F876L, induces a switch in the properties of enzalutamide from antagonist action to agonist action at the AR exchange [51], whereas T878A and L702H point mutations emerge in response to abiraterone treatment [52]. Since abiraterone is often given with prednisone (Table 1), and the L702H mutation allows for the stimulation of AR via glucocorticoids, this mutation promotes resistance to abiraterone via an enhanced sensitivity to prednisone [53,54]. AR splice variants have also been implicated in therapeutic resistance to abiraterone and enzalutamide, especially AR-V7. AR-V7 is capable of ligand-independent activation and is abundant in CRPC [11]. A landmark study by Antonarakis et al. established that AR-V7 in circulating tumor cells from patients with CRPC was associated with resistance to antiandrogens [55]. Additional mechanisms of resistance include the F877L mutation of the LBD of AR, another mutation which converts the antagonist effects of enzalutamide into agonist effects [24], and upregulation of CYP17 [53,56].

## 2. The Phenotypic Landscape of Advanced Prostate Cancer

### 2.1. Therapy-Induced Neuroendocrine Differentiation in Resistant Prostate Cancer

One mechanism of resistance that bypasses treatment to androgen axis targeting by becoming “androgen indifferent [71]” with lethal consequences is the transdifferentiation of CRPC to neuroendocrine prostate cancer (NEPC) [72]. NEPC is poorly differentiated, progresses rapidly, and metastases to visceral organs [73], with a median patient survival of 7 months [72]. It is characterized by the absence of AR [74] and the expression of neuroendocrine markers such as synaptophysin (SYP), chromogranin A (CHGA) and enolase 2 (ENO2) [73,74]. While NEPC rarely arises de novo and accounts for less than 2% of all primary prostate cancer diagnoses [71], therapy-induced NEPC (t-NEPC) can develop from divergent clonal evolution from CRPC in response to the selective pressure of ADT [72,73] and has a much higher incidence, ranging from 17–30% [73]. Furthermore, the prevalence of t-NEPC is expected to rise with the continued use of potent, second-generation antiandrogens [72]. Evidence supporting the transdifferentiation of CRPC includes the similar frequency of transmembrane protease serine 2-v ets erythroblastosis virus E26 oncogene homolog (TMPRSS-ERG) translocations in adenocarcinoma and t-NEPC [75], and the concordance of TMPRSS-ERG translocation status in the foci of both tumor types in mixed adenocarcinoma and t-NEPC [76], as well as the significant overlap in the somatic copy number alterations between CRPC and t-NEPC [77,78]. The molecular drivers of NEPC development include the cooperation between N-myc proto-oncogene protein (N-MYC) and PTEN loss or AKT1 overexpression [79], as well as AURKA (Aurora Kinase A) overexpression [76], TP53 mutation and loss of RB1 (Retinoblastoma 1) [78]. Recent work by Rotinen et al. [80] has identified the ONECUT2 (OC2) transcription factor as a targetable master regulator of lethal PCa that is also associated with the development of neuroendocrine differentiation (NED) in CRPC, as OC2 functions as a survival factor that promotes metastasis, regulates AR, and activates genes promoting neuronal differentiation in mCRPC [80]. Treatment options for t-NEPC are currently limited to platinum-based chemotherapies (cisplatin and carboplatin [72,73]), docetaxel, and etoposide [72] (a topoisomerase II inhibitor [81]), with a median survival of 7–15 months [73,82,83,84].

Taxane-based chemotherapies such as docetaxel (1st line chemotherapy) and cabazitaxel (2nd line chemotherapy) [85] are some of the only drugs available to treat advanced, metastatic prostate cancer, while novel immunotherapies [85], radioactive agents [85], and small-molecule inhibitors targeting upregulated cancer signaling pathways [86] recently provided substantial clinical promise to overcome lethal disease. Taxane chemotherapeutics exert their action on three fronts: they (a) interfere with cell division—by binding to tubulins and microtubules [87] and stabilizing microtubules from depolymerization, taxane drugs prevent cancerous cells from leaving the G2 phase and advancing to the mitotic phase of the cell cycle [88]. They also (b) promote the phosphorylation and deactivation of B-cell lymphoma 2 (BCL-2), an anti-apoptotic protein that suppresses apoptosis, by binding and inhibiting the activation of pro-apoptotic factors such as Bcl-2-like protein 4 (BAX), BCL-2 associated agonist of cell death (BAD) and BH3 interacting-domain death agonist (BID) [88], ultimately leading to apoptosis [87]. Finally, taxanes (c) inhibit AR signaling and activity as AR translocation to the nucleus is facilitated by microtubules, [89] and 1st line taxane chemotherapy downregulates AR transcriptional activity [90]. While docetaxel and cabazitaxel can improve overall survival [90], resistance eventually develops, mainly involving multidrug resistance effectors such as P-gp (P-glycoprotein) and ATP-binding cassette sub-family C member 4 (ABCC4) [91,92]. These proteins form efflux pumps that actively transport chemotherapeutic drugs out of affected cells [91,92]. Cabazitaxel has a lower affinity for P-gp compared to docetaxel, which enables it to be effective in docetaxel-resistant tumors [92,93]. Significantly enough, the antiandrogens bicalutamide and enzalutamide are also able to reduce the activity of P-gp [94] and ABCC4 [95] (Table 2).

### 2.2. Epithelial-to-Mesenchymal Transition (EMT) Navigates the Tumor Microenvironment and Contributes to Metastasis and CRPC

Impairing the emergence of therapeutically-resistant mCRPC is critical in overcoming lethal disease and improving patient survival [102,103]. Metastasis is the cause of more than 90% of cancer-related deaths, and the majority of prostate cancer-related deaths [104]. Metastasis involves the cooperation of multiple cellular processes, including the dissociation cancer epithelial cells from the primary tumor site, detachment from the extracellular matrix (ECM), resistance to anoikis, invasion, migration into blood or lymph vasculature (intravasation), circulation, extravasation, and finally, the colonization of secondary sites [102,104,105,106]. Epithelial-to-mesenchymal transition (EMT) is an essential phenotypic process functionally linked to cancer metastasis [107] as cancerous epithelial cells can reactivate and exploit the EMT program to acquire a more mesenchymal phenotype, which dramatically increases their invasiveness and metastatic potential [108]. Several transcriptional regulators of EMT in PCa, including zinc finger protein SNAI1 (SNAIL), zinc finger protein SNAI2 (SLUG), ZEB1/2, and twist-related protein 1 (TWIST) [108,109], are under the control of diverse growth factor signaling mechanisms, such as transforming growth factor-β (TGF-β) [109]. EMT is a transient reversible process, as cells with a mesenchymal phenotype can re-acquire epithelial properties (mesenchymal-to-epithelial transition (MET)), which further increases the intrigue of EMT-related effectors as potential actionable targets to impair the metastatic journey.

The prostate gland is composed of two main cell types: the glandular epithelium and the stroma [110]. Prostate carcinogenesis involves the malignant transformation of epithelial cells, supported by a reactive stroma that aids in the growth and development of the tumor [111]. The epithelial compartment contains the glandular cells of the prostate [112] and consists of a layer of basal epithelial cells that rest on a basement membrane, intermediate cells, neuroendocrine cells, and luminal epithelial cells that are secretory in nature lining the prostatic lumen [8,113,114]. Stromal tissue is connective tissue [112] and mesenchymal in origin, composed mostly of smooth muscle cells and fibroblasts [115], but also contains myofibroblasts, an extracellular matrix (ECM) composed of laminin and collagen, immune cells, nerves, and vasculature [116]. In response to androgens, and through interactions mitigated by AR [111], prostatic stroma engages in a signaling exchange with the epithelial compartment [117], which also induces the differentiation of smooth muscle cells in the stroma [118]. A multilayered forum of crosstalk between stromal and epithelial components within the prostate microenvironment contributes to tumor growth and progression to metastasis [119]. In the efferent pathway, cancer cells release soluble factors such as TGF-β and platelet-derived growth factor (PDGF) that cause changes within the stroma and activate it, known as a reactive response, whereas in the afferent pathway, cancer cells respond to and are influenced by the reactive stroma [119] towards the development of prostatic intraepithelial neoplasia (PIN) [120,121,122]. Myofibroblasts develop from their fibroblast precursors, and their phenotype is marked by the expression of vimentin, an intermediate filament that is upregulated in poorly differentiated prostate cancer and in bone metastases [123]. Myofibroblasts contribute to ECM remodeling [111,124] by secreting ECM components such as collagen I, collagen III, fibronectin isoforms, tenascin, and versican [120], as well as enzymes that help degrade the ECM—proteases such as urokinase-type plasminogen activator and matrix metalloproteases (MMPs) that cause the breakdown of basement membrane [120,124,125]. Myofibroblasts promote invasion via loss of E-cadherin [111], a transmembrane cell–cell adhesion molecule [126], and upregulation of vimentin [127] to enhance prostate tumor epithelial cell invasion and migration in metastatic prostate cancer [123].

TGF-β is a multifunctional soluble factor cytokine that has been extensively studied in prostate carcinogenesis [119] via its functional contribution to the regulation of cell proliferation, differentiation, ECM production, cell motility, migration, and apoptosis [119,128]. While part of a large superfamily of cytokines, the TGF-β subgroup consists of three isoforms [119] (TGF-β1, TGF-β2 and TGF-β3 [129]), which signal through transmembrane type I (TβRI) and type II (TβRII) receptors [119]. Signaling is initiated by the binding of activated TGF-β ligands, which bring together receptor serine/theonine kinases, the TβRI and TβRII receptors, to form a complex [130,131]. TβRII receptors activate the TβRI receptors via phosphorylation, which promotes the binding of receptor-regulated Smads (R-Smads) [130,131]. R-Smads are then phosphorylated and released from the receptor complex, where they translocate to the nucleus to bind with Smad proteins and a variety of cofactors to initiate target gene transcription [131]. Depending on ligand abundance and activity, the composition of receptor complexes, and a host of other factors, TGF-β signaling can generate hundreds of different cell-specific responses [131,132,133]. TGF-β receptor complexes may also, in certain cell types, signal through Smad-independent means, further enhancing the nuance and complexity of TGF-β signaling [131]. TGF-β can either suppress or promote tumorigenesis [134]; in early stage disease, TGF-β inhibits cellular proliferation and promotes apoptosis [128], whereas in advanced disease it functionally switches to promote metastasis [128]. This functional switch is explained by its mediation through either Smad-dependent or -independent pathways [128]. TGF-β pro-apoptotic and anti-proliferative activity is Smad-dependent and governed by Smad control of c-Myc and cyclin-dependent kinase inhibitors [128,130,135]. TGF-β signaling can also transactivate AR; Kang et al. [136] demonstrated that Smad-3, a downstream mediator in the TGF-β signaling, functions as a coregulator of AR [136]. TGF-β promotes prostate cancer progression by inducing angiogenesis and EMT [119,128], both integral processes to metastasis.

EMT and its reversible counterpart MET are critical phenotypic processes involved in embryonic gastrulation, regulation of stem cell pluripotency [137,138], remodeling of the cytoskeleton and the disruption of cell–cell adhesion and cell polarity [139]. Additionally, mesenchymal cells have an increased resistance to apoptosis and produce a higher quantity of ECM components [138]. In contrast, epithelial cells are polarized, uniform, fixed and rigid, and adhere tightly to neighboring cells and to the matrix [138,140]. The pathophysiologic byproduct of EMT related to tumorigenesis (known as Type 3 EMT; EMT related to embryo/organ development and wound healing and are denoted Type 1 and 2 EMT, respectively [140]) is that the basal surface of the epithelial cell loses adherence to its closely associated basement membrane, rendering it free to invade local structures and migrate to distant sites [140]. In doing so, these cells in transition acquire mesenchymal phenotypic markers such as vimentin and desmin [140,141]. In the context of tumor progression, EMT is not a terminal, unidirectional process that commences with epithelial cells acquiring phenotypic features that eventually make it fully mesenchymal, starting with local invasion and ending with metastasis to distant sites. Rather, cancer cells which metastasized undergo phenotypic reversal to their epithelial traits, such re-expressing E-cadherin [138], in order to colonize secondary sites [140] through MET. The bidirectional nature of EMT-MET is an attractive therapeutic target [142] to impair metastasis.

EMT is characterized by the loss of the transmembrane cell–cell adhesion molecule E-cadherin, as well as occludins and claudins that are essential to maintaining the integrity of a stable epithelium [126,138,143]. This loss of E-cadherin is under multitier regulatory control in both physiologic and pathophysiologic EMT [140,144,145]. At the transcriptional level, repressors such as SNAIL [146], SLUG [147], ZEB family [148], and TWIST [149] downregulate the expression of E-cadherin through binding to the E-box region of the E-cadherin promoter [150]. While these transcription factors coordinate to upregulate the expression of one another in a complex pattern [151], their overall activation is mediated by TGF-β [140,151]. Decreased E-cadherin is often succeeded by N-cadherin, a protein that increases the migratory and invasive capacity of tumor cells [138,152]. E-cadherin loss directly promotes metastasis by facilitating the dissociation of cancer cells from the tumor mass [145,153]. Post-translationally, E-cadherin is anchored to the actin cytoskeleton as part of a complex with β-catenin [138,154]. β-catenin is a protein that serves multiple functions, one of which is as a critical component in the canonical Wnt signaling pathway [154,155], which plays a role in embryologic development, stem cell maintenance, and when mutated or dysregulated, contributes to tumorigenesis [156]. Loss of E-cadherin renders β-catenin free to translocate to the cytoplasm and participate in the Wnt signaling cascade [157]. β-catenin can also bind to cytosolic AR, which upon translocation into the nucleus increases AR transcriptional activity [138]. Of clinical relevance are the associations between co-expressed AR/β-catenin and Gleason grade 4–5 tumors and higher PSA levels [158], and the enrichment of AR and Wnt signaling in patients with early stage cancer [159].

Metastasis and colonization of distant sites by cancer cells requires overcoming anoikis, or detachment-induced cell death [102]. Cancerous cells must lose their adhesive properties and be able to detach from the ECM, evade apoptosis, and have the capacity for anchorage-independent growth at secondary sites [102]. As cells detach, anoikis is activated via the ECM-integrin cell survival pathway and by the mitochondrial mediated pathway [102]. Resistance to anoikis promotes prostate tumor migration, invasion, and metastasis [108]. Drivers of this event include the overexpression of galectin proteins (especially Galectin-3) [108,160], the activation of TRrkB (a neurotrophic tyrosine kinase) with its ligand brain-derived neurotrophic factor (BDNF), the upregulation of caveolin-1, and an increase in IGF-1 signaling [108].

### 2.3. Integrated Targeting of Non-Coding RNAs with EMT to Overcome Therapeutic Resistance in Advanced Prostate Cancer

Non-coding RNAs (ncRNAs) are molecules that are transcribed but not translated into protein products, serving the function of altering gene expression at the transcriptional, translational, and post-translational levels [161,162]. Non-coding RNAs are generated from intergenic sequences, from the introns of protein-coding genes or from antisense strands [161], and are broadly characterized by size as either small (<200 nucleotides) or long (>200 nucleotides) ncRNAs [161]. Non-coding RNAs function as regulatory molecules that mediate a wide array of cellular processes such as chromatin remodeling, transcription and post-transcriptional modifications [163], and as such, certain ncRNAs are known to be capable of functioning as oncogenes or tumor suppressors [164]. As it is estimated that over 90% of the human genome encodes for non-protein coding RNAs, and that close to 75% of those genes encode for ncRNAs [165], it is reasonable to assume that ncRNAs play a far more important—and far more complex—role in regulating gene expression in cancer than we currently realize. Excitingly, several ncRNAs have been seen to hold tremendous potential, or already serve as diagnostic or prognostic biomarkers for PCa, while other ncRNAs appear highly attractive as targets for therapeutic intervention [161]. Small non-coding RNAs include microRNAs (miRNAs), PIWI-interacting RNAs (piRNAs), small nuclear RNAs (snRNAs), small nucleolar RNAs (snoRNAs), and transfer RNA-derived small RNAs (tsRNAs), among others [164,166,167], while long non-coding RNAs (lncRNAs) include antisense RNAs, sense intronic RNAs, pseudogenes and circular RNAs (circRNAs) [164,166,168]. In PCa, evidence is continuing to mount which reveals the role that several kinds of both small and long ncRNAs have in regulating EMT and metastasis.

MicroRNAs are short (19–25 nucleotide) ncRNAs that regulate post-transcriptional gene expression by either targeting mRNAs for cleavage or by repressing their translation, interacting with the 3′- untranslated regions (UTRs) of target mRNAs [169,170,171]. They are among the most extensively studied and well-known of the ncRNAs in cancer and have repeatedly been implicated for their roles in regulating EMT in PCa [172,173]. MicroRNAs can be oncogenic or tumor-suppressive, and regulate EMT in PCa by either directly inhibiting EMT-related transcription factors or cytoskeletal components or by regulating the signaling pathways involved in EMT [173]. The miR-200 family of miRNAs (miR-200a, miR-200b, miR-200c, miR-141, and miR-429) are important negative regulators of metastasis via EMT inhibition that are all downregulated in PCa [173]. Thus, miR-200 inhibits ZEB1, ZEB2, and SLUG expression in PC3 cells [173,174]. In another study, Liu et al. had observed that both miR-1 and miR-200b target SLUG, and that SLUG also acts as a repressor of miR-1 and miR-200b transcription, suggesting that SLUG and miR-1/miR-200b regulate one another in a feedback loop that amplifies EMT [173,175]. Importantly, the overexpression of either miRNA leads to decreased SLUG, reduced growth and invasion and the reversal of EMT to MET in human PCa cell lines [173,175]. Reversing EMT via the reintroduction of EMT-suppressive miRNAs has been observed by other investigative groups, including work performed by Basu et al., wherein Hsa-miR-200c was overexpressed in hypoPC3 cells and was found to reverse the EMT markers vimentin, ZEB1, and SLUG [176]. The overexpression of miR-21 in PC3 cells leads to increased expression of vimentin and N-cadherin, and the downregulation of E-cadherin [173,177]. More recent studies [178] identified two onco-miRNAs, 181a-5p and 181b-5p, as contributors to migration and invasion inhibitory protein (MIIP), a protein with tumor suppressor functions [178]. MIIP was shown to inhibit these two miRNAs, which normally act to suppress KLF17 [178], a protein that has been shown to inhibit EMT in other human cancers [179,180]. MIIP inhibition of miRNA 181a-5p and 181b-5p also leads to reduced expression of SNAIL and TWIST, and knockdown of MIIP promoted tumor growth or osteolytic bone lesions [178], as well as AR activity [181]. Specifically, miRNA-299, located at chromosome 14q32.31, is one of a large cluster of miRNAs that have been implicated in the progression of prostate cancer [182,183]. The overexpression of miR-299 was shown to inhibit the expression of SLUG and TGF-β3 and increase E-cadherin expression, correlating with low AR levels and consequential reduced cell migration and proliferation [182]. Another emerging protagonist is miRNA-539, which was found to act by inhibiting the expression of the oncogene DLX1, leading to downstream reduction in levels of SNAIL, SLUG and vimentin, as well as inhibiting the TGF-β/Smad axis [184]. Other miRNAs linked to EMT markers inhibit the invasion of prostate cancer cells through the TGF-β/Smad axis, including miR15a/16 [185], as the overexpression of miR15a/16 leads to decreased invasiveness of LNCaP cells and the downregulation of SNAIL and TWIST [185]. Excitedly, some miRNAs have been seen to have a positive effect on overcoming therapeutic resistance, as miR27-b and miR34-a suppression of ZEB1 was shown to correlate with overcoming docetaxel resistance in vitro [186].

#### Extracellular Vesicles Deliver Critical Cargo

Exosomal miRNAs (exomiRNAs) have also been linked to EMT in PCa. Exosomes are a group of extracellular vesicles that were discovered in 1969 by H.C. Anderson, and have long been studied for their role in the intercellular transport of small molecules and trafficking within the tumor microenvironment [187,188]. Exosomes are produced by all cell types, range in size from 40 nm–150 nm and contain DNA, RNA and proteins [189]. Exosomal miRNAs are among the cargo of these vesicles that have been studied for their role in cancer progression [190], including PCa [191]. Exosomal miR-26a was found to downregulate N-cadherin and vimentin, while increasing E-cadherin levels in LNCaP cells [192]. Another exosomal miRNA, miR-1246, was found to be downregulated in prostate cancer cells, but was found to inhibit tumor growth in vivo while inhibiting N-cadherin and vimentin levels in vitro, suggesting a protective role against disease progression for this miRNA [193].

**Figure 1:**
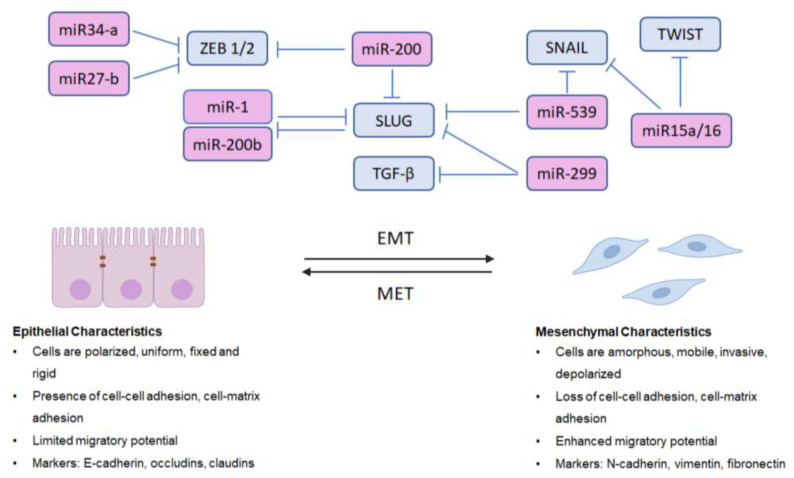
microRNA (miRNA) Regulation of Epithelial-to-Mesenchymal Transition (EMT) in Cancer. Over 30 different miRNAs are now known to interact with EMT-related signaling effectors in prostate cancer. This schema reveals the critical miRNAs that negatively regulate EMT, via inhibition of SNAIL, SLUG, ZEB1/2, TWIST and TGF-β. Regulation of EMT is complex, as miRNAs can have multiple targets (miR-299, miR15a/16) or are themselves regulated by EMT components in a feedback loop (miR-1/miR-200b and SLUG). The process of EMT involves the cellular transformation from an epithelial phenotype to a mesenchymal one in preparation for metastasis. Utilizing miRNAs and other non-coding RNAs (ncRNAs) to regulate EMT interconversion to mesenchymal-to-epithelial transition (MET) in order to impair metastasis could be a novel therapeutic strategy to treat advanced or resistant prostate cancer.

As there are at least 30 different miRNAs known to interact with EMT pathways and components in PCa [173], not all can be described in detail here; however, it is clear that miRNAs are emerging as key regulators of EMT in PCa. A schematic illustration of significant miRNAs that negatively regulate phenotypic EMT in PCa is shown in Figure 1.

P-element-induced wimpy testis (PIWI)-interacting RNAs (piRNAs) are a relatively new class of ncRNA, having been discovered in 2006; however, there is some evidence to suggest their role in contributing to EMT in prostate cancer [194]. These small ncRNAs are single-stranded, 24–32 nucleotides in length and interact with PIWI-proteins to form piRNA-PIWI complexes, which are involved in germline development, stem cell maintenance, epigenetic regulation and translation control [194,195,196]. In cancer, overexpression of PIWI proteins PIWIL1 and PIWIL2 has been observed in several types of carcinoma, including breast, esophageal, gastric, ovarian and colorectal cancers [194]. In prostate cancer, Yang et al. observed increased expression of PIWIL2 in malignant prostate specimens compared to non-malignant adjacent tissues [194,197], while the silencing of PIWIL2 led to decreased cell invasion and migration in PC3 prostate cancer cells [194,197]. Furthermore, loss of PIWIL2 impacted EMT effectors, reduced the expression of N-cadherin, TWIST, and vimentin, and increased E-cadherin levels [194,197]. Of translational significance is evidence that a three-piRNA signature (hsa_pir_000627, hsa_pir_005553 and hsa_pir_019346) is associated with clinical biochemical recurrence [198]. More recently, using small RNA sequencing Zhang et al. found two piRNAs, piR-001773 and piR-017184, to be upregulated in PCa, their expression correlating with Gleason score and pathological stage [199], and increased expression of piR-001773 and piR-017184 promoted the invasion and migration of androgen-independent prostate cancer cells [199]. Thus, compelling evidence supports the regulatory role of PIWI-piRNA complexes and piRNAs in EMT, with enhanced clinical relevance in PCa (Figure 1).

Small nucleolar RNAs (snoRNAs) are small (60–300 nucleotide) ncRNAs that are actually processed intron fragments, essentially recycled by cells to be used in pre-RNA processing [200,201]. SnoRNAs typically form complexes with catalytic ribonucleoproteins and function as guide RNAs that base-pair with the complementary RNA sequences that are to be modified [200,201]. SnoRNAs are usually found within the nucleoli, where they are responsible for post-transcriptionally modifying and maturing many other types of RNA, and are divided into two classes depending on the types of modifications they perform (C/D box for 2′-*O*-ribose methylation and H/ACA box for pseudouridylation) [201]. SnoRNAs have in recent years become increasingly implicated in tumorigenesis and cancer, with evidence building in support for their roles in contributing to EMT in prostate cancer and prostate cancer progression [202]. Sequencing of the small RNA transcriptome of normal and malignant tissues revealed that snoRNA-derived RNAs (sdRNAs) are highly upregulated in PCa and account for over one third of the differentially expressed small RNAs in tumor tissue compared to normal adjacent tissues [203]. SdRNAs derived from C/D box snoRNAs were also found to be associated with the development of metastatic disease [203]. In a separate study, Crea et al. had found that SNORA55 was upregulated in metastatic vs. non-metastatic paired PCa xenografts, and that it could also predict shorter relapse-free survival [203]. Silencing of SNORA55 led to reduced proliferation and migration in PCa cell lines [204]. In 2018, Yi et al. found that H/ACA snoRNA SNORA42 was upregulated in PCa cell lines and tissue samples, and that the overexpression of SNORA42 inhibited apoptosis and increased cell proliferation, migration and invasion [202]. Additionally, PC3 and DU145 cells transiently-transfected with SNORA42 were found to have increased expression of vimentin, *N*-cadherin and ZEB1 with decreased expression of E-cadherin, while small interfering RNA (siRNA) knockdown of SNORA42 led to a reversal of this phenotype, with decreased vimentin, N-cadherin and ZEB1, paralleled by an increased expression of E-cadherin [202].

Long non-coding RNAs (lncRNAs, those ncRNAs that are >200 nucleotides in length) are another major class of ncRNAs known to be involved in regulating EMT and prostate cancer progression. They are structurally similar to protein coding genes in several respects, yet they possess no open reading frames, have fewer exons and are generally expressed at lower levels than their protein coding counterparts [161,164]. Compared to smaller ncRNAs, lncRNAs are able to fold into secondary and tertiary structures [162] and exhibit far greater functional diversity [164]. LncRNAs can regulate gene expression at the epigenetic, transcriptional, and post-transcriptional levels, and can either operate near their own sites of transcription (i.e., *cis*-acting) or act in distant genomic or cellular locations relative to where they were transcribed (i.e., *trans*-acting) [164]. Their regulatory mechanistic repertoire includes the ability to guide chromatin modifiers to specific genomic locations (to activate or suppress transcription), alter pre-mRNA splicing, inhibit mRNA translation, and act as decoys to displace transcriptional repressors or as scaffolds for multiple protein complexes to interact with one another [205,206]. One of the first lncRNAs to be described in PCa was prostate cancer gene expression marker 1 (PCGEM1), a lncRNA that inhibits apoptosis and promotes cell proliferation in vitro via enhanced androgen-dependent gene transcription [161]. Amongst the lncRNAs most characterized as clinically relevant is prostate cancer antigen 3 (PCA3), a unique, atypically alternatively spliced lncRNA mapped to the long arm of human chromosome 9q21–22 [207] and overexpressed in >95% of primary prostate tumors [161,208]. PCA3 is the most specific prostate cancer molecule currently known to date, and is used as a diagnostic biomarker for PCa in the US, Europe and Canada [207]. Functional loss of PCA3 increases the expression of SLUG, SNAIL, and E-cadherin in LNCaP cells [209]. Some lncRNAs act by competitively binding to miRNAs, while others act independently of miRNAs. Specifically, ZNFX1 antisense RNA 1 (ZFAS1) [210] and small nucleolar RNA host gene 3 (SNHG3) [211] have been shown to bind miRNAs that inhibit EMT and promote the apoptosis of prostate cancer cells. LncRNA SNHG7 was also suggested to promote EMT in prostate cancer via binding to miRNA-324-3p, as well as through the Wnt pathway [212], while the lncRNA E3 ubiquitin-protein ligase (CHFR) was found to act through multiple pathways via miR-10b to promote EMT in PC3 cells, mainly through the GSK/AKT and NF-κB pathways [213]. In oral squamous cell carcinoma, the downstream targets of lncRNAs include the PI3K/AKT pathway, under the regulation of lncRNA metastasis associated lung adenocarcinoma transcript 1 (MALAT1) [214]. In the same study, it was also shown that MALAT1 modulation of the PI3K/AKT pathway was associated with EMT induction [214]. In prostate cancer, the loss of MALAT1 impedes the growth of PCa xenografts [215] and reduces cell proliferation and migration, while it promotes apoptosis in AR-negative prostate cancer cells [216]. VIM antisense RNA 1 (VIM-AS1) increases N-cadherin and vimentin while downregulating E-cadherin in promoting prostate cancer EMT [217].

Circular RNAs (circRNAs) have also been linked to EMT and PCa progression, although the evidence supporting these roles for circRNAs in PCa is continuing to emerge. Circular RNAs are closed loop sequences of RNA that lack 5′ or 3′ ends, and have the ability to affect gene expression by binding to miRNA (acting as miRNA sponges), RNA binding proteins, and protein kinases, among other components [218]. Dai et al. found that the circRNA myosin light chain kinase (MYLK) was significantly upregulated in both bladder and prostate cancers, and that it promoted cancer progression via the downregulation of miRNA-29a expression [219]. In PCa, circular RNA17 has been found to be inversely correlated to prostate cancer aggressiveness and enzalutamide resistance [220]. One circRNA, circSMAD2, plays a role in attenuating EMT in prostate cancer cells (Figure 1). Han et al. demonstrated that circSMAD2 levels were low in prostate cancer cells and that circSMAD2 upregulation led to the inhibition of invasion and EMT through miR-9 [221].

### 2.4. Epigenetic Regulation by ncRNAs Contributes to EMT and Disease Progression

Epigenetic modifications are diverse, and include covalent modifications to DNA (i.e., acetylation, methylation, phosphorylation) as well as post-translational modifications to histones [206,222]. An altered epigenetic landscape both results from and contributes to cancer, a landscape that can be actively shaped from the participation of ncRNAs [206]. Dysregulated ncRNA expression is associated with the development of tumors and can influence epigenetic modifications; however, interestingly enough, ncRNA dysregulation appears to primarily result from epigenetic changes [206].

MicroRNA regulation of the epigenome occurs via their post-transcriptional silencing of epigenetic modifiers such as histone deacetylases (HDACs), histone methyltransferases (HMTs) and DNA methyltransferases (DNMTs) [206]. An important example of miRNA epigenetic regulation in prostate cancer is miR-101 regulation of enhancer of zeste homolog 2 (EZH2) [223]. EZH2 is a catalytic subunit that is part of the chromatin-modifying, epigenetic modulator polycomb repressor complex 2 (PRC2), and is overexpressed in PCa and associated with metastatic and neuroendocrine disease [223,224,225]. In fact, EZH2 is thought to be a master regulator of NEPC reprogramming and is overly expressed in the vast majority (87%) of NEPC patients [225]. miR-101 negatively regulates EZH2, and the downregulation of miR-101, which is frequently seen in PCa, may be directly responsible for the upregulation of EZH2 [223,226]. Functional restoration of miR-101 expression inhibits EZH2 and decreases cell proliferation and tumor invasiveness [223,226]. Mechanistically, the expression of members of the miR-200 family (including miR-205) that regulate EMT in PCa are themselves epigenetically regulated [223]. miR-205 expression, for example, is downregulated in PCa via hypermethylation of its promoter, and is associated with resistance to chemotherapy. Significantly, miR-200c and miR-141 are also downregulated in androgen-independent prostate cancer cells and contain a hypermethylated CpG promoter, but not in androgen-sensitive cells, in which the promotor region of these genes remains unmethylated [223,227,228].

LncRNA epigenetic regulation also occurs through their direct interactions with epigenetic modifiers [206]. The lncRNA second chromosome locus associated with prostate-1 (SChLAP1) is found to be overexpressed in PCa, with significantly increased levels in metastatic tumors [206,229]. Mechanistically, SChLAP1 interacts with and antagonizes SWItch/Sucrose Non-Fermentable (SWI/SNF), a chromatin remodeling complex that exhibits tumor-suppressive activity, thus SChLAP1 overexpression promotes cell invasion and metastasis [206,229]. The lncRNA HOXD cluster anti-sense RNA 1 (HOXD-AS1) is also overexpressed in PCa and highly expressed in CRPC cells, and correlates with Gleason score and metastasis [230]. Recent mechanistic insights revealed that HOXD-AS1 recruits WD repeat-containing protein 5 (WDR5), a key subunit of the lysine-specific methyltransferase 2A (MLL1) chromatin remodeling complex, and regulates target gene transcription via mediating histone H3 lysine 4 tri-methylation (H3K4me3) to promote chemo-resistance of human prostate cancer cells [230].

The concerted involvement of so many various noncoding RNAs and other molecular species in epigenetic gene regulation can be utilized to generate clinically-useful epigenetic noncoding RNA signatures with prognostic or diagnostic value [231]. While few to no epigenetic biomarkers exist that can identify aggressive phenotypes, epigenetic biomarkers are emerging that can, for example, predict clinically significant cancer in patients on active surveillance (AS) [231]. The development and progression of PCa are frequently associated with epigenetic changes such as global DNA hypomethylation, and the hypermethylation of genes such as GSTP1 (glutathione S-transferase Pi 1) and HOXD8, and the dysregulation of ncRNAs such as miR-129a (decreased expression) and miR-18a (increased expression) [231]. Table 3 summarizes current evidence supporting the functional contributions of ncRNAs to EMT and defining their potential clinical value as biomarkers in prostate cancer progression.

### 2.5. Pharmacologic Targeting of EMT to Overcome Prostate Cancer Resistance

There has been extensive investigation into various therapeutic actionable targets in signaling pathways EMT to MET interconversions. Cajigas-du Ross et al., [246] for example, performed RNA-seq on docetaxel-resistant and -sensitive prostate cancer cells and found that E-cadherin levels were significantly reduced, while there was an increase in vimentin, SNAIL and TWIST levels in docetaxel-resistant prostate cancer cell lines [246]. Moreover, the same group reported robust upregulation of tetraspanin-8 (TSPAN8) as one of several key proteins involved in this process, which encodes proteins involved with cell–cell communication via interaction with integrins [246], suggesting its involvement in the acquisition of chemoresistance in PCa. Xue et al. [247] treated human androgen-independent prostate cancer cells with a combination of zinc and paclitaxel in an attempt to overcome taxane resistance [247]. Zinc was found to increase prostate cell sensitivity to paclitaxel, while the combination of zinc and paclitaxel decreased the expression of TWIST1 and induced apoptosis [247]. Loss of TWIST1 increased the sensitivity of these cells to taxane [247], while increased ZEB1 was detected in response to docetaxel; taken together, this evidence implicates a role for EMT regulators in resistance to taxane chemotherapy [248].

Receptor tyrosine kinases (RTKs) have also been studied for their connection to EMT and taxane resistance in prostate cancer. One such RTK, tyrosine-protein kinase receptor UFO (AXL), has been implicated for its role in resistance to imatinib and erlotinib in leukemia and non-small cell lung cancer, respectively [249,250]. In prostate cancer, AXL was found to be overexpressed in docetaxel-resistant cell lines, and AXL overexpression alone was found sufficient to induce resistance to docetaxel [251]. The inhibition of AXL abated EMT phenotypic features and suppressed tumor proliferation and migration, positing AXL as a possible therapeutic target to overcome docetaxel resistance [251].

The PI3K/AKT survival signaling pathway has also been implicated in shaping the EMT phenotypic landscape within the prostate tumor microenvironment. Chen and colleagues probed the PI3K/AKT pathway using the tumor suppressor inositol polyphosphate 4-phosphatase B (INPP4B) on prostate cancer cells, finding that overexpression of INPP4B led to increased sensitivity to docetaxel [252]. Mechanistically, INPP4B was found to inhibit the PI3K/AKT pathway, as well as upregulate E-cadherin and reduce levels of vimentin, fibronectin, and N-cadherin [252], thus the PI3K/AKT pathway could be a link between docetaxel resistance and EMT. Additionally, pre-clinical models have demonstrated that splice variants of AR, most notably AR-V7, are linked to EMT and mesenchymal phenotypes [253,254]. The EMT transcriptional suppressor SNAIL enables a potential link between full-length AR, AR splice variants and EMT, as increasing levels of SNAIL promote antiandrogen resistance and increased AR activity, whereas the repression of SNAIL re-sensitized resistant prostate cancer cells to enzalutamide [255].

The anoikis-driven antitumor effect of α1-adrenoreceptor antagonists promises a safe-strategy in treating advanced disease—both therapeutically-resistant and castration-sensitive prostate cancer [143,256,257]. Quinazoline-based compounds developed after the pharmacological optimization of α1-adrenoceptor antagonists cause phenotypic reversion of EMT to MET and induce anoikis towards overcoming resistance to AR antiandrogens in pre-clinical models of advanced prostate cancer [143,257,258,259].

## 3. Conclusions

Since the original work by Charles Huggins in 1941 on the effects of ADT on progression to lethal disease, the emergence of castration resistance in patients with prostate cancer has reinforced the need for understanding actionable drivers of prostate cancer progression beyond AR, its ligands, and downstream targets. Prostate cancer is remarkably heterogenous and driven by a host of molecular factors; evidence-based knowledge of the genomic and molecular underpinnings of PCa has paved the way for personalized treatments and reliable biomarkers with diagnostic or prognostic value. The PARP (poly (adenosine diphosphate (ADP)-ribose) polymerase) inhibitor olaparib and the lncRNA biomarker PCA3 mentioned previously are two such examples. Olaparib, originally used to treat BRCA-driven ovarian cancers [260], was recently FDA approved last year for the treatment of mCRPC in men with alterations in genes involved in homologous recombination repair who failed antiandrogen therapy [70]. PARP is an enzyme involved in multiple DNA repair pathways and in repairing single strand breaks, which eventually lead to cell death if not addressed [261]. Interestingly, and fittingly so, recent mechanistic evidence revealed that the silencing of PARP1 in prostate cancer cells suppresses their growth and induces MET [262].

Non-coding RNAs are as rich and diverse in function as they are in number, and intense efforts pursue their potential to become clinically actionable. One could easily argue that defining the role of each ncRNA as a driver of phenotypic EMT in the context of prostate cancer metastasis and/or therapeutic resistance would lead to the development of novel phenotypic or molecular signatures that yield diagnostic and therapeutic value (summarized on Table 3). Could ncRNA signatures better predict NEPC, therapeutic resistance or metastasis-free survival? Considering the emerging value of ncRNA biomarkers, these genomic regulators are of high potential clinical relevance as prostate-specific signatures with therapeutic targeting value in lethal disease.

## Figures and Tables

**Table 1:** Clinical Significance of Current Treatment Modalities for Advanced Prostate Cancer: Results from Clinical Trials. Clinical efficacy of current treatment modalities for advanced prostate cancer based on results from clinical trials.

Class	Drug	Clinical Significance
Antiandrogens	Apalutamide (Erleada^®^)	TITAN: In patients with mCSPC, improved OS and PFS compared with placebo [57] SPARTAN: In patients with nmCRPC, improved MFS compared to placebo [58]
	Enzalutamide (Xtandi^®^)	AFFIRM: In patients with mCRPC who failed docetaxel, improved radiographic PFS and OS [47] PREVAIL: In patients with mCRPC who had no prior chemotherapy, improved radiographic PFS and OS [48] PROSPER: In patients with nmCRPC, improved time to metastatic progression or time to death compared with placebo [59]
	Darolutamide (Nubequa^®^)	ARAMIS: In patients with nmCRPC, improved metastasis-free survival compared with placebo [60]
	Abiraterone acetate (Zytiga^®^)	COU-AA-301: In patients with mCRPC after docetaxel, abiraterone + prednisone improved median PFS and OS compared to placebo + prednisone [61] COU-AA-302: In chemotherapy-naïve patients with mCRPC, abiraterone + prednisone improved median radiographic PFS and OS compared to placebo + prednisone [61] LATITUDE: In patients with newly diagnosed high risk CRPC, abiraterone + prednisone + maintenance of traditional ADT improved median radiographic PFS and OS compared with traditional ADT alone [62]
Anthracyclines	Mitoxantrone + prednisone	In patients with symptomatic mCRPC, improved symptoms without difference in OS compared with prednisone alone [63]
Taxanes	Docetaxel (Taxotere^®^)	TAX 327: In chemotherapy-naïve patients with mCRPC, improved OS in docetaxel dosed every 3 weeks compared with weekly docetaxel and mitoxantrone dosed every 3 weeks [64] SWOG 9916: In patients with mCRPC, docetaxel + estramustine improved OS compared with mitoxantrone + prednisone [65]
	Cabazitaxel (Jevtana^®^)	TROPIC: In patients with mCRPC who progressed after receiving docetaxel, improved OS and PFS compared with mitoxantrone [66]
Immunotherapy	Sipuleucel-T (Provenge^®^)	IMPACT: In patients with asymptomatic or minimally symptomatic mCRPC (most were chemotherapy-naïve and did not have visceral metastases), improved OS compared with placebo [67]
	Pembrolizumab (Keytruda^®^)	KEYTRUDA: FDA approved in 2017 for patients with microsatellite instability-high or mismatch repair-deficient tumors deemed unresectable or metastatic, including patients who have failed prior treatments [68,69]
PARP inhibitors	Olaparib (Lynparza^®^)	PROfound: In patients with mCRPC who received enzalutamide or abiraterone, with homologous recombinant repair genetic alterations (BRCA1, BRCA2, ATM), delayed disease progression [70]

**Table 2:** Current Treatment Modalities for Advanced Prostate Cancer and their Mechanisms of Action. Pharmacological targeting of advanced prostate cancer. Pharmacological targeting of advanced prostate cancer: hormone-sensitive prostate cancer (HSPC), castration-resistant prostate cancer (CRPC), and metastatic CRPC.

Class	Drug	Disease Stage	Mechanism of Action
Antiandrogens	Apalutamide (Erleada^®^)	nmCSPC [96]	Blocks AR → inhibits AR translocation to nucleus and prevents binding to DNA [12]
	Enzalutamide (Xtandi^®^)	nmCRPC mCRPC with prior chemotherapy mCRPC without prior chemotherapy [96]	Blocks AR → inhibits AR translocation to nucleus and prevents binding to DNA [12]
	Darolutamide (Nubequa^®^)	nmCRPC [97]	Blocks AR → inhibits AR translocation to nucleus and prevents binding to DNA [12]
	Abiraterone acetate (Zytiga^®^)	CSPC mCRPC with prior chemotherapy mCRPC without prior chemotherapy [96]	Inhibits CYP17 → Prevents biosynthesis of androgens [6,12]
Anthracyclines	Mitoxantrone + prednisone	Symptomatic CRPC	Inhibits DNA topoisomerase II → Disrupts DNA synthesis and repair [98]
Taxanes	Docetaxel (Taxotere^®^)	mCRPC [96]	Stabilizes microtubules → inhibits depolymerization → G2M arrest [88] Phosphorylates and deactivates BCL-2 → BCL-2 unable to inhibit pro-apoptotic factors (e.g., BAX, BAD and BID [88]) Impairs nuclear translocation of AR [89]
	Cabazitaxel (Jevtana^®^)	mCRPC with prior docetaxel [96]	Stabilizes microtubules → inhibits depolymerization→ G2M arrest [88] Reduced affinity for p-glycoprotein [92]
Immunotherapy	Sipuleucel-T (Provenge^®^)	Minimally symptomatic mCRPC [96]	Autologous CD54+ dendritic cells processed into fusion protein with PAP and GM-CSF, given as vaccine to generate immune response in prostate microenvironment [99,100]
	Pembrolizumab (Keytruda^®^)	Metastatic microsatellite instability-high or mismatch repair deficient solid tumors with progression on prior treatments and no other treatment options [96]	Anti-PD1 monoclonal antibody [68]
PARP inhibitors	Olaparib (Lynparza^®^)	mCRPC with germline or somatic mutations in BRCA1/BRCA2 or ATM [96]	Inhibits PARP, an enzyme involved in DNA repair [101]

**Table 3:** Diagnostic and Prognostic Biomarker Potential of Several EMT-Associated ncRNAs in Prostate Cancer. Several non-coding RNAs functionally involved in prostate cancer EMT have potential diagnostic and/or prognostic clinical value.

ncRNA	Functional Involvement in EMT	Molecular Target(s)	Clinical Relevance	Potential Diagnostic and Prognostic Value
**miRNAs**				
miR-141	Inhibits and/or reverses EMT [232] miR-141 overexpression inhibits metastatic potential, decreases vimentin, fibronectin and increases E-cadherin [232]	NF-κB signaling (TRAF5 and TRAF6) [232]	miR-141 found significantly upregulated in metastatic patients, as well as in patients with PCa compared to those with BPH or healthy donors [233,234]	Diagnostic [233,234]
miR-200b	Inhibits and/or reverses EMT [235] miR-200b overexpression decreases tumor growth, invasion and metastasis [235]	ZEB1 and ZEB2 [235]	Highly expressed in metastatic disease [233,234]	Diagnostic [233,234]
miR-375	Inhibits and/or reverses EMT [236] miR-375 overexpression increases Zona occludens-1 (ZO-1), decreases vimentin, fibronectin, invasion and migration [236]	YAP1 [236]	Highly expressed in metastatic disease and associated with poor OS [233,234,237]	Diagnostic [233,234]
miR-1246	Inhibits and/or reverses EMT [193] miR-1246 overexpression decreases EMT and cell proliferation, invasion and migration [193]	N-Cadherin and Vimentin [193]	miR-1246 can distinguish between healthy donors and patients; highly expressed in aggressive tumors and lymph node metastases (selectively released in exosomes) [193]	Diagnostic/prognostic [193]
let-7c	Inhibits and/or reverses EMT [238,239] let-7c overexpression decreases cell proliferation, and anchorage-independent growth [240]	HMGA1, HMGA2, MYC, BCL2, Caspase-3 [239,241]	Expression of let-7c associated with advanced clinical stage [234,238]	Prognostic [234,238]
**lncRNAs**				
HOTTIP	Promotes EMT [242] HOTTIP overexpression increases cell proliferation, invasiveness and migration [242]	miR-216a-5p [242]	High levels associated with poor clinicopathologic features [243] and lymph node metastases [242]	Prognostic [243]
SNHG12	Promotes EMT [244] SNHG12 overexpression increases cell proliferation, invasiveness and migration [244]	miR-195 [244] and miR-133b [245]	Highly expressed in tumor and associated with Gleason score and lymph node metastases [244]	Prognostic [245]
**snoRNAs**				
SNORA42	Promotes EMT [202] SNORA42 overexpression increases cell proliferation, invasion and migration [202]		High expression associated with disease progression [202]	Prognostic [202]

## Abbreviations

ABCC4	ATP-binding cassette sub-family C member 4
ADT	Androgen Deprivation Therapy
AKT	Protein kinase B
AKT1	RAC-alpha serine/threonine-protein kinase
AR	Androgen Receptor
AR-V	AR splice variant
AR-V7	AR splice variant 7
AS	Active surveillance
AXL	Tyrosine-protein kinase receptor UFO
AURKA	Aurora Kinase A
BAD	BCL-2 associated agonist of cell death
BAX	Bcl-2-like protein 4
BCL-2	B-cell lymphoma 2
BD	Brain-derived neurotrophic factor
BID	BH3 interacting-domain death agonist
BPH	Benign prostatic hyperplasia
CHFR	E3 ubiquitin-protein ligase
CHGA	Chromogranin A
circRNA	Circular RNA
CRPC	Castration-resistant prostate cancer
CSPS	Castration-sensitive prostate cancer
CYP17	Cytochrome p450 enzyme 17R-hydroxylase-17,20-lyase
DHEA	Dehydroepiandrosterone
DHT	Dihydrotestosterone
DLX1	Homeobox protein DLX-1
DNA	Deoxyribonucleic acid
DNMT	DNA methyltransferase
ECM	Extracellular matrix
EGF	Epidermal growth factor
EMT	Epithelial-to-mesenchymal transition
ENO2	Enolase 2
ExomiRNA	Exosomal miRNA
EZH2	Enhancer of zeste homolog 2
GM-CSF	Granulocyte-macrophage colony-stimulating factor
GSTP1	Glutathione S-transferase Pi 1
H3K4me3	Histone H3 lysine 4 tri-methylation
HDAC	Histone deacetylase
HER2/neu	Human epidermal growth factor receptor 2
HMT	Histone methyltransferase
HOXD-AS1	HOXD cluster anti-sense RNA 1
IGF-1	Insulin-like growth factor 1
IGFBP-3	Insulin-like growth factor-binding protein 3
INPP4B	Inositol polyphosphate 4-phosphatase B
KGF	Keratinocyte growth factor
KLF17	Kruppel Like Factor 17
LBD	Ligand binding domain
LH	Luteinizing hormone
LHRH	Luteinizing hormone releasing-hormone
lncRNA	Long non-coding RNA
MALAT1	Metastasis associated lung adenocarcinoma transcript 1
mCRPC	Metastatic castration-resistant prostate cancer
mCSPC	Metastatic castration-sensitive prostate cancer
MET	Mesenchymal-to-epithelial transition
MFS	Metastasis-free survival
MIIP	Migration and invasion inhibitory protein
miRNA	microRNA
MLL1	Lysine-specific methyltransferase 2A
MMP	Matrix metalloprotease
mTOR	Mammalian target of rapamycin
MYLK	Myosin light chain kinase
N-MYC	N-myc proto-oncogene protein
ncRNA	Non-coding RNA
NED	Neuroendocrine differentiation
NEPC	Neuroendocrine prostate cancer
nmCRPC	Non-metastatic castration-resistant prostate cancer
nmCSPC	Non-metastatic castration-sensitive prostate cancer
OC2	ONECUT2 transcription factor
OS	Overall survival
P-gp	P-glycoprotein
PAP	Prostatic acid phosphatase
PARP	Poly (adenosine diphosphate (ADP)-ribose) polymerase
PCa	Prostate cancer
PCA3	Prostate cancer antigen 3
PCGEM1	Prostate cancer gene expression marker 1
PD1	Programmed cell death protein 1
PDGF	Platelet-derived growth factor
PFS	Progression-free survival
PI3K	Phosphoinositide 3-kinase
PIN	Prostatic intraepithelial neoplasia
piRNA	PIWI-interacting RNAs
PIWI	P-element-induced wimpy testis
PIWIL1	Piwi-like protein 1
PIWIL2	Piwi-like protein 2
PRC2	Polycomb repressor complex 2
PSA	Prostate specific antigen
PTEN	Phosphatase and tensin homolog
R-Smad	Receptor-regulated Smad protein
RB1	Retinoblastoma 1
RNA	Ribonucleic acid
RTK	Receptor tyrosine kinase
SChLAP1	Second chromosome locus associated with prostate-1
sdRNA	SnoRNA-derived RNA
siRNA	Small interfering RNA
SLUG	Zinc finger protein SNAI2
SNAIL	Zinc finger protein SNAI1
SNHG3	Small nucleolar RNA host gene 3
SNHG7	Small nucleolar RNA host gene 7
snoRNA	Small nucleolar RNA
snRNA	Small nuclear RNA
SWI/SNF	SWItch/Sucrose Non-Fermentable
SYP	Synaptophysin
t-NEPC	Therapy-induced NEPC
TβRI	Type I transforming growth factor-β receptor
TβRII	Type II transforming growth factor-β receptor
TGF-β	Transforming growth factor-β
TGF-β2	Transforming growth factor-β2
TGF-β3	Transforming growth factor-β3
TMPRSS-ERG	Transmembrane protease serine 2-v‑ets erythroblastosis virus E26 oncogene homolog
TP53	Tumor protein 53
TrkB	Tropomyosin receptor kinase B
TSP-1	Thrombospondin 1
TSPAN8	Tetraspanin-8
tsRNA	Transfer RNA-derived small RNAs
TWIST	Twist-related protein 1
UTR	Untranslated region
VEGF	Vascular endothelial growth factor
VIM-AS1	VIM antisense RNA 1
WDR5	WD repeat-containing protein 5
ZEB1	Zinc finger E-box-binding homeobox 1
ZEB2	Zinc finger E-box-binding homeobox 2
ZFAS1	ZNFX1 antisense RNA 1
ZO-1	Zona occludens-1
